# From Oxidized Fatty Acids to Dimeric Species: In Vivo Relevance, Generation and Methods of Analysis

**DOI:** 10.3390/molecules28237850

**Published:** 2023-11-29

**Authors:** Jenny Leopold, Patricia Prabutzki, Kathrin M. Engel, Jürgen Schiller

**Affiliations:** Institute for Medical Physics and Biophysics, Faculty of Medicine, Leipzig University, Härtelstr. 16-18, D-04107 Leipzig, Germany; patricia.prabutzki@medizin.uni-leipzig.de (P.P.); kathrin.engel@medizin.uni-leipzig.de (K.M.E.); juergen.schiller@medizin.uni-leipzig.de (J.S.)

**Keywords:** dimeric fatty acids, FAHFA, fatty acids, (lipid) oxidation, hypochlorous acid (HOCl), mass spectrometry

## Abstract

The occurrence of free fatty acids (FFAs) and the generation of reactive oxygen species (ROS) such as hydroxyl radicals (HO^●^) or hypochlorous acid (HOCl) is characteristic of inflammatory diseases, for instance, rheumatoid arthritis. Unsaturated fatty acids react with ROS yielding a variety of important products such as peroxides and chlorohydrins as primary and chain-shortened compounds (e.g., aldehydes and carboxylic acids) as secondary products. These modified fatty acids are either released from phospholipids by phospholipases or oxidatively modified subsequent to their release. There is increasing evidence that oligomeric products are also generated upon these processes. Fatty acid esters of hydroxy fatty acids (FAHFAs) are considered as very important products, but chlorinated compounds may be converted into dimeric and (with smaller yields) oligomeric products, as well. Our review is structured as follows: first, the different types of FFA oligomers known so far and the mechanisms of their putative generation are explained. Industrially relevant products as well as compounds generated from the frying of vegetable oils are also discussed. Second, the different opinions on whether dimeric fatty acids are considered as “friends” or “foes” are discussed.

## 1. Introduction

Many diseases are accompanied by inflammatory processes, which are connected to complex metabolic processes. This does not only apply to classical inflammatory diseases such as arthritis, hepatitis or gastritis but is also valid for other diseases such as cancer [1]. One of the first steps in inflammation is the infiltration of typical inflammatory cells including (neutrophilic) granulocytes, macrophages or T-cells [2]. After their activation, these cells release a variety of different enzymes with tissue-destroying capability (such as elastase and collagenase) as well as reactive species (RXS) like reactive oxygen (ROS), halogen (RHS) and nitrogen (RNS) species. RXS (ranging from low-reactive O_2_^●−^ and H_2_O_2_ to more reactive compounds such as hypochlorous acid (HOCl) and, particularly, hydroxyl radicals (HO^●^)) [3] are presumably the most relevant for further oxidation processes and, thus, the progress of inflammation. Here, we focus on ROS- and RHS-generated dimeric oxidized products evolved from unsaturated lipids including free fatty acids (FFAs) [4].

**Figure 1:**
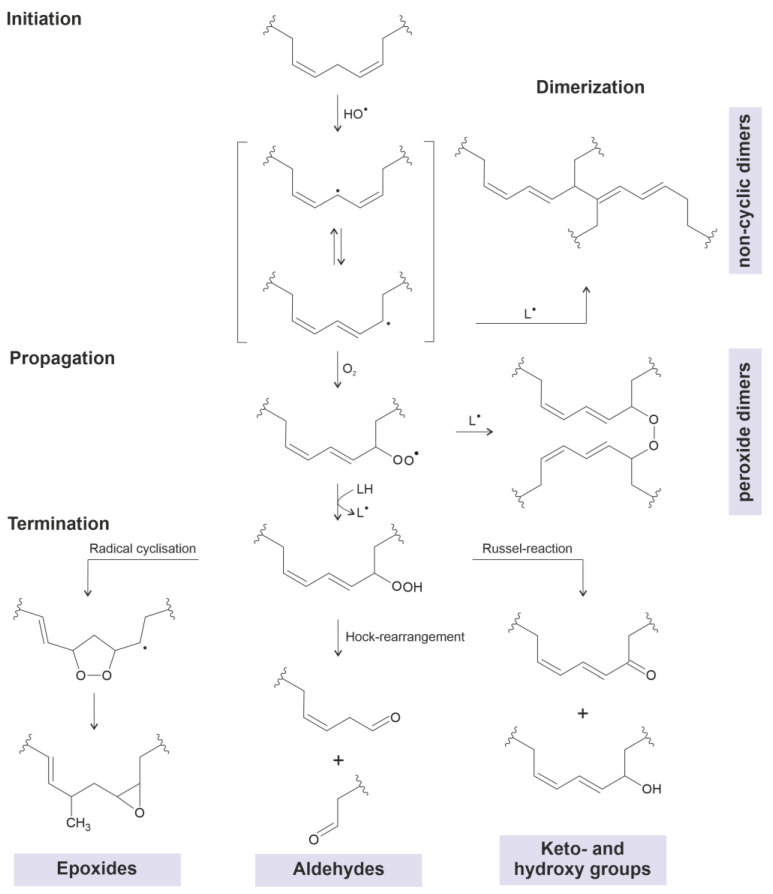
Scheme of potential lipid oxidation products generated from ROS. Oxidized lipids may either dimerize directly after the initiation via radical reactions or after the termination into chain-shortened oxidized products like carboxylic acids, aldehydes, ketones or hydroxylated products. All shown oxidation products are able to dimerize via different mechanisms which are discussed here. Abbreviations: HO^●^: hydroxyl radical, LH: linoleic acid (as selected example), L^●^: radical of linoleic acid.

It is commonly accepted that inflammatory processes lead to the gradual modification of virtually all biomolecules. Aside from the functional groups of some amino acids (particularly the sulfur-containing ones methionine and cysteine), the double bonds in lipids are also affected by selected ROS and RNS [5]. The related reactions of (unsaturated) lipids with ROS are of considerable interest due to the high abundance of lipids in many tissues and body fluids. After the initial addition of oxygen to the double bond, a cleavage at this position may occur as a second step, leading to chain-shortened products such as aldehydes [6] or carboxylic acids [7]. In addition to this reaction, hydroxylated fatty acids may be generated [8]. The introduction of the reactive hydroxyl group [9] is an important prerequisite of many further reactions since the hydroxyl group is capable of reacting with free carboxylic acids. A survey of potential oxidation products is summarized in Figure 1.

Esterification is a well-known chemical equilibrium reaction at which the equilibrium is on the side of the educts, i.e., the alcohol and the acid. Boiling under reflux for several hours (normally with the removal of the generated water) is necessary to shift the equilibrium to the product side, i.e., to the ester. This is no major problem for in vitro studies in the laboratory but requires the presence of suitable enzymes in biological systems and/or catalysts in chemical systems. Since the formation of fatty acid dimers attracts increasing interest in both industrial and (patho-)physiological aspects, the corresponding dimeric fatty acid compounds are discussed in this review (vide infra).

Our paper is structured according to the increasing complexity of the evolving dimeric species from technically relevant fatty acid dimers to heat-induced changes of vegetable oils (upon cooking and/or frying) to the in vivo relevance of fatty acid dimers and the most relevant species known so far. The focus will be on branched-chain fatty acid hydroxyl fatty acids (FAHFAs), and both potential generation mechanisms and disease-related aspects will be highlighted. Finally, we will shortly introduce our own research work and illustrate that HOCl (an ROS; sometimes, also called RHS or even RCS (reactive chlorine species) generated by inflammatory cells in the presence of the enzyme myeloperoxidase) leads to the conversion of oleic acid into a variety of dimeric (and in minor yields oligomeric) chlorohydrin products in vitro.

## 2. Industrial Relevance of Fatty Acid Dimers

Although there is increasing biological and physiological interest in dimeric fatty acids, these compounds represent technically relevant chemicals, too. Produced in the ton scale, dimeric fatty acids (often generated by the dimerization of oleic (18:1; denotation: X:Y; number of carbon atoms:double bond content) or linoleic (18:2) acid) are important in the production process of biodegradable plastics [10] as lubricants [11] and adhesives [12]. Since these fatty acid dimers are not readily generated (for instance by a 4 + 2 cycloaddition), a catalyst is required to improve the reaction yield. Montmorillonite (MMT) [13], a natural mineral with a defined layered structure (a natural phyllosilicate), is an efficient dimerization catalyst to convert common fatty acids into dimers. MMT is by far the most widely used catalyst in industry to obtain dimeric fatty acids, with ongoing attempts to improve the efficiency of this material as recently summarized in [14]. This paper also provides a comprehensive summary of the generated products and their structures.

**Figure 2:**
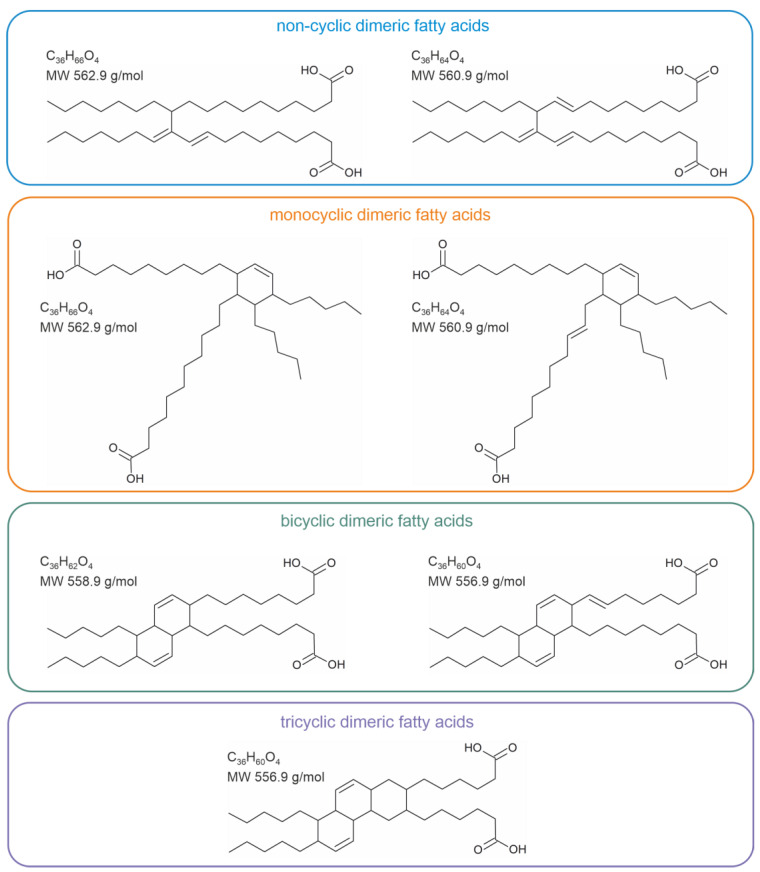
Different technically important products, which can be expected upon the reaction of fatty acids (with at least one double bond) with each other in the presence of a suitable catalyst. This figure is based on [14] with slight modifications.

It is important to note that these industrial dimeric fatty acids are coupled exclusively via C-C bonds without the presence of any heteroatoms (Figure 2). Dimeric fatty acids are commonly synthesized under the exclusion of atmospheric oxygen but in the presence of a suitable catalyst at temperatures of about 230 to 250 °C. At these rather harsh reaction conditions, complex product patterns arise (Figure 2) with a variety of products in different yields.

Although these product mixtures are commercially available in large amounts at moderate prizes, the separation and structural characterization of the individual compounds is extremely challenging due to their structural similarities. This particularly applies if fatty acid mixtures are subjected to polymerization. Thus, there are considerable efforts to impact the yields of the desired product [15].

In order to characterize the complex mixtures of fatty acid multimers generated through industrial processes, several analytical techniques are available. Despite the challenging differentiation of the individual isomers, there are methods available that enable at least the reliable separation of the monomers, dimers and trimers [16]. Normal-phase (silica gel) thin-layer chromatography (TLC) using *n*-hexane, cyclohexane and diethyl ether (55:15:5, *v*/*v*/*v*) as the eluents was suggested as the method of choice to obtain reasonable separation quality [16]. In an older study, different analytical methods (with the focus on chromatography) were compared to analyze dimeric fatty acids [16]. Although many different methods are basically suitable to overcome the analytical challenges, only a handful of them have so far actually been applied. However, the capabilities of TLC, gas chromatography (GC) and liquid chromatography (LC)—often coupled to mass spectrometry (MS)—to investigate mixtures of different dimeric fatty acids were comprehensively compared [15]. The outcome of this study was that high-performance liquid chromatography (HPLC) is the optimum method to separate complex mixtures. This statement is even valid nowadays, and HPLC-MS is the method of choice for a relatively fast and convenient dimer fatty acid (and FAHFA) analysis (vide infra) [17]. Nevertheless, great care is needed to avoid potential misinterpretations [18]. This particularly applies if HPLC is combined with MS detection since some ions may be exclusively generated in the gas phase and, thus, falsely indicate the “real” presence of some products.

## 3. Heat-Induced Dimerization of Vegetable Oils

One important point with the direct nutritional aspects is the generation of oligomeric (oxidation) products during the deep-frying of food [19]. It is important to note that vegetable oils with many double bounds (such as sunflower or linseed oil) are normally not used for frying. Vegetable oils, such as coconut oil [20] and palm oil, which contain mainly saturated fatty acyl residues are commonly used and exposed to heat (about 250 °C) for prolonged times in the presence of atmospheric oxygen with the aim to prepare typical fried dishes. These harsh conditions lead to thermal stress and the generation of a variety of (lipid) oxidation products. A detailed discussion of all these compounds would exceed the size of this review. Instead, interested readers are referred to the excellent studies of Koelmel and colleagues [21] and Choe and Min [22]. The exact frying chemistry depends on a number of different parameters including but not limited to [23]:

- The double content of the triacylglycerols (TAG) within the oil;
- The frying temperature;
- The time of frying;
- The (residual) water content of the oil;
- The presence of transition metals which are known to foster the decomposition of initially generated lipid hydroperoxides [24];
- The presence of vitamins and natural antioxidants such as tocopherols [25].

**Figure 3:**
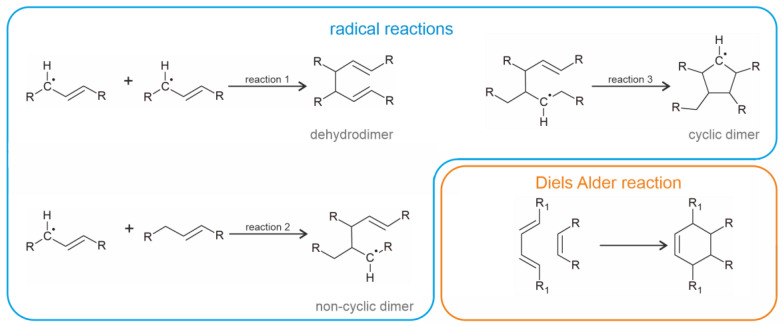
Suggested products for the generation of dimeric fatty acids during thermal stress of unsaturated triacylglycerols (TAGs) in vegetable oils. Note the 2 + 4 cycloaddition during the Diels Alder reaction.

Suggested mechanisms to explain the generation of selected dimeric compounds in heated vegetable oils (according to [26,27]) are shown in Figure 3.

The majority of mechanistic investigations are dedicated to unsaturated TAGs. Molecular oxygen is quickly added to the unsaturated fatty acyl residues within the oil [28]. The initially generated peroxides subsequently decompose into more stable but transient products such as aldehydes, which are capable of reacting with the (amino group-containing) food under the generation of potential harmful products [23]. Since the released aldehydes (such as acrolein or hydroxynonenal) are particularly harmful, their detection is attracting increasing interests [29,30]. A survey on the products that arise in thermally stressed oils is available in [26].

Very recently, it was shown that these products, often summarized as the “peroxide value”, are ligands of the transcription factor PPARγ [31]. Although the discussion of these aspects is beyond the scope of this review, it must be stated that even saturated TAGs (that are normally recommended for the frying of food due to their comparably low reactivity) may undergo pronounced oxidative changes. A mechanism explaining this reaction was previously suggested [20].

More relevant to the topic of this review is the observation that oligomeric products are formed as a consequence of the frying process [32]. These are of the initially generated peroxyl radicals which are transient products. This aspect was only recently investigated in more detail [33]. Nevertheless, the structural evaluation of the fatty acid dimers, or even the TAG dimers, is still a challenge due to the structural similarities of the generated products [26,34]. The mechanism of the TAG hydrolysis is also not completely clear yet since water is required to induce the hydrolysis of the fatty acids (reverse reaction of the ester condensation) [35]. Frying oil should contain, however, only trace amounts of water since the oil is heated far above the boiling point of water (≈250 °C vs. 100 °C). Potential oxidized TAG products are summarized in Figure 1, and the suggested reaction mechanisms for the dimerization processes of these oxidized TAGs are shown in Figure 2.

Suitable analytical methods to monitor the different products are summarized in [36,37]. Chromatographic techniques (HPLC and GC) allow for the separation between apolar TAG species and more polar oxidatively-modified TAG monomers and polymers in general [36]. MS, either in combination with LC [38] or as a shotgun approach, is the method of choice to monitor the presence of virtually all potential oxidation products. Both matrix-assisted laser desorption/ionization (MALDI) [39] as well as electrospray ionization (ESI) [21] are MS techniques that have been successfully used. Although MALDI MS is more convenient and enables a high sample throughput, it has one severe weakness: labile compounds such as hydroperoxides cannot be accurately analyzed since they decompose into more stable compounds when they are subjected to laser irradiation [40]. Therefore, information about labile compounds is lost. On the contrary, the use of a semi-targeted reversed-phase LC-MS approach, while more laborious, enables the in-depth analysis of multiple oxidation products in complex mixtures [38]. A recently published review focuses on the methodological aspects to analyze food lipids and dedicates a section to oxidized lipids in food, too [41].

Nuclear magnetic resonance (NMR) is also a widely used method to investigate technically relevant dimers as well as for the monitoring of oxidation products in thermally stressed vegetable oils [42]. The majority of these studies rely on ^1^H NMR because ^1^H is the most sensitive nucleus and is present to a considerable extent in all organic compounds. There are some characteristic chemical shifts, for instance, that of the aldehyde group in thermally stressed oils in the range of 9 to 10 ppm. NMR is also the method of choice to confirm the presence of monounsaturated fatty acid ether oligomers formed during the heating of virgin olive oil [43]. Another study focused on the ^1^H NMR of cyclic compounds derived from fatty acid dimerization with a proton on the cyclic ring showing a particular chemical shift of δ = 6.8 ppm [44].

^31^P NMR has a much broader range of chemical shifts than ^1^H NMR but is much more sensitive compared to ^13^C NMR. Additionally, no solvent suppression (H_2_O) is required. ^31^P NMR is increasingly being used to investigate the presence of minor components in vegetable oils. Of course, it does not detect common fatty acids due to the lack of phosphorus. To overcome this, 2-chloro-4,4,5,5-tetramethyldioxaphospholane was established [45] as a suitable derivatization reagent to introduce the required phosphorus atom into acidic compounds. This phospholane reacts with all acidic protons, for instance, those in hydroxyl groups that can subsequently be detected. This makes the method suitable for the detection of small amounts of oxidation products in vegetable oils [46]. Since no major laboratory work is needed, this method is very elegant and convenient. However, further work is required to check whether dimeric species are also detectable using ^31^P NMR.

## 4. Fatty Acid Dimers with In Vivo Relevance

As already indicated above, FAHFAs can be generated by the reaction of the carboxyl group of an unmodified fatty acid with the hydroxyl group of a hydroxylated fatty acid, followed by an intermolecular (i.e., between two different molecules) ester condensation. The FAHFA research was first initiated about 10 years ago with the first paper published in 2014 [47]. Due to the importance of this field, there are already many timely reviews, for instance [48,49,50].

**Figure 4:**
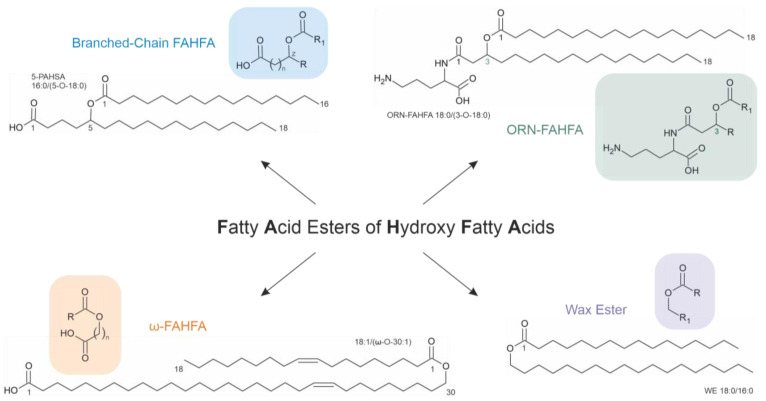
General structures (highlighted in color) and one example for each of the four fatty acid ester of hydroxy fatty acid (FAHFA) families: branched-chain FAHFA, ornithine (ORN)-FAHFA, omega (ω)-FAHFA and wax esters (WEs).

There are four main FAHFA families: branched-chain FAHFA, omega (ω)-FAHFA, ornithine (ORN)-FAHFA and wax esters (WEs) [48] (Figure 4). Every family comprises many different subclasses and structural isomers, which makes the detailed FAHFA analysis challenging.

### 4.1. Branched-Chain FAHFA

Branched-chain FAHFAs (also called estolides) are endogenous lipids formed through the acylation of medium-chain (regularly C16–C18) hydroxyl fatty acids [49] with many metabolic (such as antidiabetic and anti-inflammatory—but not necessarily limited to these) effects. Each subclass consists of an ester of different acyl chains and multiple isomers with branch points at different carbon atoms. For instance, 5-PAHSA (Figure 4) evolves from the reaction of 5-hydroxy-stearic acid with the carboxyl group of palmitic acid. In general, all hydroxy fatty acids may also undergo an intramolecular reaction between the hydroxyl and the carboxyl groups under the generation of a lactone. However, such products only seem to play a major role if shorter fatty acids (with 8–12 carbon atoms) are considered [51]. The structural variability of FAHFAs is remarkable. For instance, Zhu and colleagues identified 51 different FAHFA subclasses comprising more than 300 isomers in mouse visceral adipose tissue. The authors found that the number of potential isomers is age-dependent [52].

The chemical synthesis of estolides is uncommon but also possible. Castor oil, a vegetable oil pressed from castor beans, contains 90% ricinoleic acid (12-hydroxy-9-*cis*-octadecenoic acid), a highly relevant compound for industry. Castor oil is, therefore, a promising compound for estolide synthesis. Furthermore, an intramolecular ester condensation (lactone generation) might occur. Such lactones can be synthesized in the laboratory [53] but do not seem to play a role under natural conditions. There are (to the best of our knowledge) no reports on the endogenous presence of FAHFAs in castor oil. Nevertheless, a review on the chemical synthesis of FAHFAs has recently been published [54].

Palmitic-hydroxystearic (PAHSA) and palmitic-hydroxypalmitic (PAHPA) subclasses have so far been the focus of research. They include the 5-, 7-, 8-, 9-, 10-, 11-, 12- and 13-hydroxy regioiomers, i.e., they possess a significant variability [17]. While these FAHFAs are de novo synthesized in many organs, they are also present in common food plants such as oat, clementine, garlic and pineapple [55]. Estolides possess some interesting properties—particularly, they are considered as promising drugs [17,50].

Since the presence of one hydroxy group is mandatory for the biosynthesis of branched-chain FAHFAs, the in vivo production of FAHFAs can be increased through oxidative stress (for instance, by the use of an inhibitor of glutathione synthesis as a common antioxidant) [56]. An in vivo FAHFA biosynthesis comprises the transfer of a fatty acyl residue from a fatty acyl-CoA to a hydroxy fatty acid via lipid enzymes termed acyltransferases [57], particularly palmitoyl acyltransferases. In contrast, the catabolism (i.e., in vivo degradation) of FAHFAs relies on threonine and serine hydrolases as well as a carboxyl ester lipase, which both hydrolyze the FAHFAs into the free fatty acid and the corresponding hydroxy fatty acid [58]. Enzymes are mandatory for degradation: the chemical hydrolysis of FAHFAs under acidic conditions (as in the stomach during the digestion process) is not sufficient to induce a FAHFA hydrolysis.

The question of whether FAHFAs (due to their structural similarity to FFAs) can be incorporated into other lipid classes like diacylglycerols (DAG) has been recently addressed [59]. The resulting FAHFA-containing TAGs (FAHFA-TAGs; with a somewhat uncommon residue in the *sn*2 position) are an important storage pool for branched-chain FAHFAs [59,60]. There is one remarkable issue regarding this lipid class: FAHFA-TAG concentrations were about 100 times higher than those of free FAHFAs. This might indicate that FAHFA-TAGs act as a storage pool of FAHFAs in cells and tissues. Details of the generation of these lipids (including the involved enzymes) were recently elucidated [61].

Branched-chain FAHFAs might act as therapeutic targets to fight against metabolic diseases such as type II diabetes, hepatic steatosis, cardiovascular diseases or even some kinds of cancer [62] because they represent efficient, endogenous anti-inflammatory [47] and anti-diabetic (regulators of glucose homeostasis) [54,63] lipids. Nevertheless, FAHFAs may also exhibit severe side effects. For instance, selected FAHFAs induced hepatic steatosis and fibrosis in mice [64]. In 2020, Zhu et al. discovered an age dependency of the FAHFA concentration in the visceral adipose tissue of mice [52]: FAHFA concentrations were rather abundant in middle-aged mice but less abundant in both younger and older mice. The authors concluded that FAHFAs might be relevant for catabolic processes in growth, development and aging. As an example of anti-inflammatory effects, 13-LAHLA was found to suppress the lipopolysaccharide-stimulated secretion of cytokines and the expression of genes with pro-inflammatory effects [65]. A recent review on this topic is recommended to the interested reader [48].

Branched-chain FAHFAs are also known to be constituents of oils such as fish, corn, palm, soybean and olive oil. The highest concentration of 12-OAHSA, particularly, was monitored in olive oil [66]. FAHFAs are present in the majority of oils in concentrations of about 8–29 pmol/mg oil [67]. This study found FAHFA 36:3, FAHFA 36:2 and FAHFA 36:4 in nut oil, as well as FAHFA 36:2, FAHFA 34:1 and FAHFA 36:1 in olive oil; FAHFA 32:1, FAHFA 34:0, FAHFA 36:0 and FAHFA 36:1 were detected in all common vegetable oils [67]. From the described beneficial effects of FAHFAs to human health, oil consumption can be, thus, recommended even in low-caloric diets.

Although details are still widely unknown, there is a feedback mechanism, i.e., the FAHFA concentration is directly influenced by fasting as well as high-fat feeding, as seen in volunteers, which emphasizes the in vivo importance of FAHFAs [PMID: 34322511]. Particularly, LAHLAs are not exclusively characteristic of animals but also occur to some extent in plants [65], for instance, oat, where all FAHFAs together may reach µg quantities. It is of interest to note that some oat oil fractions and, particularly, FAHFA-enriched extracts from the respective fractions exhibited significant anti-inflammatory activity [55]. Three LAHLA isomers (15-, 13- and 9-LAHLA) represented the most abundant FAHFAs in the oat oil fraction. Native linoleic acid (18:2) possesses its double bonds in positions 9 and 12. Thus, the occurrence of 13-LAHLA can be explained by a shift of the double-bond position (likely induced by oxidation) from Δ12 into Δ13 [65]. Also, 13-LAHLA was the most abundant LAHLA isomer in human serum subsequent to the consumption of liposomes from oat oil [65]. In contrast to vertebrates, mono- (MGDG) and digalactosyldiacylgylcerols (DGDG) are characteristic lipid constituents of plants and algae [68]. Therefore, it is not surprising that DGDG-monoestolides were detected in oats [69].

Using different assays, it became evident that PAHSA bind to the G protein-coupled receptor 40 (GPR40). This is accompanied by the increased glucose stimulation of the insulin release [70]. In human white adipose tissue (WAT), 583 FAHFA isomers out of 21 FAHFA subclasses could be successfully monitored [50]. Due to the different classes, there is a large number of potential fatty acyl residues and large structural similarities but only small in vivo concentrations (about 10 pmol/mg protein in tissues) [71]. Analytical aspects are of paramount importance in FAHFA research. This particularly applies to the question of the extent to which the length of the acyl residues plays a role. High-resolution MS is the method of choice for the evaluation of suitable fragment ions in tandem-mass (MS/MS) spectra and enables the differentiation between the fatty acid and the hydroxy fatty acid but does not enable unequivocal identification of the position of the hydroxyl group [47]. Therefore, high-resolution chromatography [72] and/or ion mobility spectroscopy [73] are essential in order to resolve the different isomers (identical molecular weights). There are targeted and non-targeted approaches. The non-targeted approach is easier and more convenient to perform in the first place and less time-consuming since direct infusion (without previous separation) into the mass spectrometer can be used. However, great care is required to avoid potential misinterpretations due to missing chromatographic separation. For instance, it was recently shown [18] that artifactual fatty acid dimers, which are generated in the gas phase (particularly if relatively concentrated solutions are investigated), may mimic FAHFA signals. These artifacts arise when a deprotonated and a neutral fatty acid collide with each other in the gas phase. Such mis-assignments are a particular problem if mass spectrometers with a poor resolution are used. Under these conditions, it is not possible to differentiate isobaric ions, which may lead to false identifications.

It is important to know that the released fragments possess different intensities and the fatty acids possess higher intensities than the hydroxylated fatty acids. There is also a computer-generated library with MS/MS spectra for more than 1000 FAHFA species available [74]. Using high-resolution tandem MS with nanoelectrospray ionization, FAHFA-TAGs were recently investigated in detail (i.e., the position of the FAHFAs and the positions of modification were successfully elucidated) [60].

### 4.2. ω-FAHFA

Omega (ω)-FAHFAs (Figure 4) are generated when a very-long-chain ω-hydroxy fatty acid (30 to 34 carbons) reacts with a free fatty acid [49]. These FAHFAs represent biological surfactants in tears [75], amniotic fluid [76], sperm [77] and some other body fluids. Although the relevance of this finding is not yet known, ω-FAHFAs were exclusively found in the head and not in the tail of sperm and could play a role in the fertilization process [77].

Pathways leading to the required ω-hydroxyl fatty acids are still not yet clearly defined. Most of the related studies have focused on bacteria [78]. From a chemical point of view, it is particularly amazing that ω-FAHFAs do not contain intramolecular esters, which should be generated (compared to intermolecular products) if highly diluted (in vivo) samples are investigated: under these conditions, there is a low probability that two different compounds will collide with each other. This is, however, one prerequisite of a successful reaction.

In selected cases (such as vernix caseosa or meibum), compounds with a cholesteryl moiety next to classical ω-FAHFAs have been described [79]. These compounds are not very abundant but can be easily assigned by the characteristic loss of *m*/*z* 369 in the positive-ion-mode ESI mass spectra (proton adduct of cholesterol subsequent to water loss) [80].

### 4.3. Ornithine-FAHFA

To the best of our knowledge, these compounds have so far been exclusively detected in some selected bacteria [81], particularly in Gram-negative bacteria, where they are also known to stabilize the lipid membrane. Thus, stress to the bacteria is accompanied by an increased production of these compounds. A bacterial ornitho-FAHFA biosynthesis requires the N-acylation of ornithine with a 3-hydroxy fatty acid. This reaction is catalyzed by a particular enzyme (ORN lipid O-acyltransferase A) [82] which is missing in vertebrates. Further details (with a focus on bacterial lipidomics) are available in [83].

### 4.4. Wax Esters

WEs represent esters of a saturated fatty acid and a saturated fatty alcohol (Figure 4). Carnauba (from the leaves of the Brazilian carnauba palm), candelilla (from the leaves of the small Candelilla shrub in Central America) and beeswax are the most important commercial waxes. In addition to their occurrence in these matters, WEs are also abundant in some bacteria and other prokaryotes, which is discussed in detail in [84].

WEs do also occur in mammals and were generated through a classical ester condensation under the need of a fatty alcohol. In the first step, the enzyme fatty-acyl-CoA reductase converts a fatty acyl-CoA into the required fatty aldehyde. In the next reaction step, this aldehyde is subsequently converted into the fatty alcohol by the enzyme fatty aldehyde reductase. The ester condensation is mediated by the enzyme wax alcohol acyltransferase. WEs occur in mammals in the sebum [85], skin [85], hair [86] and saliva [87]. Although they are considered important because of their lubricating properties, there are so far no reports about the importance of WEs as diagnostically relevant molecules, but they are highly relevant in cosmetics.

## 5. Dimeric Fatty Acids Generated by HOCl

According to the currently available knowledge, dimeric fatty acids are generated either through Diels–Alder reactions [88] between a monounsaturated fatty acid and linoleic acid or—more abundantly—through the secondary reactions of oxidized fatty acids [56]. The latter applies particularly to thermally stressed vegetable oils [36]. However, there are also some more recent suggestions for the generation of the dimers [89].

HOCl is an important oxidant that is generated from Cl^−^ and H_2_O_2_ under the catalysis of the enzyme myeloperoxidase (MPO) [90], which is a characteristic protein of inflammatory cells, i.e., it represents about 5% of the total amount of proteins in neutrophils. In comparison to radical oxidants such as hydroxyl radicals, HOCl is a “molecular oxidant” and leads, thus, to the generation of defined products. HOCl also shows a defined order of reactivities, i.e., thiol groups are most rapidly modified followed by aromatic residues and amino groups. Although the reactivity of olefinic residues (double bonds) with HOCl is comparably poor, unsaturated fatty acids as well as (phospho)lipids are converted into chlorohydrins as the most abundant products [90].

**Figure 5:**
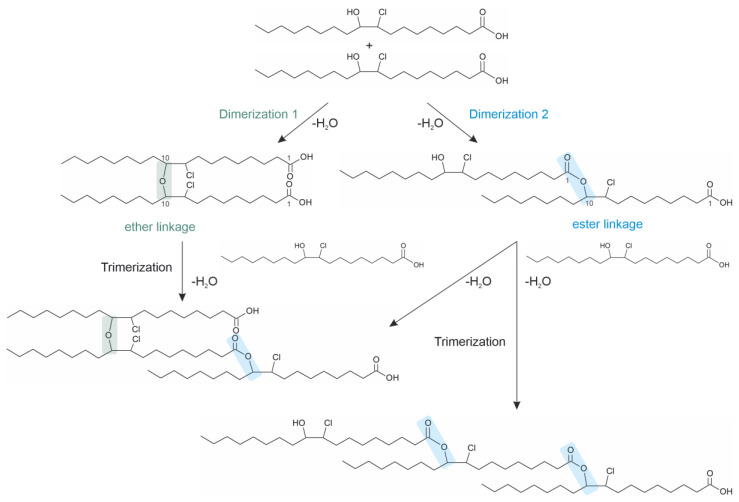
Putative mechanisms by which dimeric and trimeric species are generated from oleic acid chlorohydrins in vitro [89]. In the first step, oleic acid is oxidized by hypochlorous acid (HOCl) via electrophilic addition of Cl^+^ (electrophile) to the double bond in the fatty acid, followed by the nucleophilic addition of the hydroxyl ion to the positively-charged intermediate. As a result, two isomeric chlorohydrins are generated (here, only one isomer is shown for both oleic acid chlorohydrins). In a second step, two chlorohydrins dimerize either via esterification leading to an ester linkage or under the formation of an ether bond between the two hydroxyl groups. By addition of another fatty acyl chlorohydrin, even a trimeric species could be formed via the formation of an ether or ester bond.

The chlorohydrin formation is described by an electrophilic addition of Cl^+^ (from HOCl) as an electrophile to the double bond of unsaturated molecules, leading to the formation of a carbocation and the subsequent addition of OH^−^ as a nucleophile. The two generated chlorohydrin isomers seem to oligomerize either via ester condensation or under the formation of an ether bond between the two hydroxyl groups (Figure 5). Thus, both estolide analoga and ether species of chlorohydrins could be generated even under physiological conditions in vitro (pH 6.5, 37 °C) [89].

Is has to be mentioned that there are only selected reaction products of oleic acid shown in Figure 5. The appearance of potential isomers is neglected, and the figure is only used to demonstrate the dimer and trimer formation of fatty acid chlorohydrins. The product yield is even higher when polyunsaturated fatty acids are oxidized with HOCl, resulting in oligomeric products.

Chlorohydrins can be easily detected in biological mixtures because they undergo a relatively slow enzymatic degradation and, thus, accumulate in biological fluids [91]. Studies using different chromatographic methods such as high-performance thin-layer chromatography (HPTLC) have provided evidence that there are also oligomeric (mainly dimeric) products generated [89]. Some selected products of oleic acid chlorohydrins are shown in Figure 5. Monomeric oleic acid chlorohydrins can already be detected in mice and humans suffering from acute pancreatitis using GC-MS [92,93]. The detection of FFA chlorohydrins and its oligomers with MALDI-TOF MS is difficult, since at least the monomeric generated ions are partially suppressed by the signals of the MALDI matrix, which is present in excess compared to the analyte. Thus, the obtained product pattern should be characterized using ESI MS with or without previous chromatographic separation [89].

## 6. Summary and Outlook

**Figure 6:**
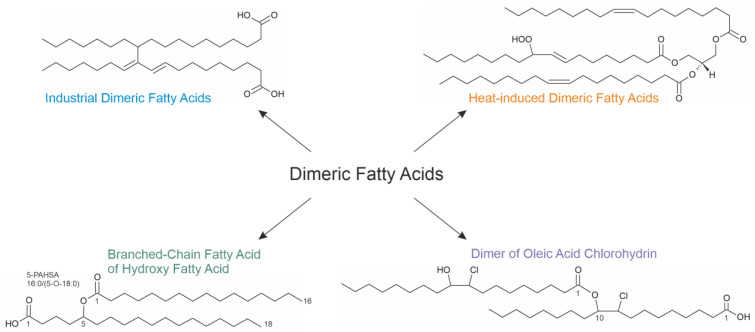
Structures of the discussed dimeric fatty acid types with industrial, nutritional or in vivo relevance. All shown structures are only examples of a multitude of different products. In the case of the heat-induced products, a transient product is shown, which is subsequently converted into dimeric products.

Fatty acid-derived compounds have been known for many years to be involved in various physiological processes and to be important in human health [94]. In this short review, we have discussed different oligomeric species of native fatty acids from which some examples are summarized in Figure 6:

We have discussed four different types of dimeric fatty acid species industrially synthesized, generated via frying processes or generated from fatty acids in vivo. The four different types could be summarized as follows:

1. The first compounds (industrial dimeric fatty acids) are generated in the absence of oxygen when fatty acids are heated in the presence of a suitable catalyst. They do not contain oxygen bridges but are linked via C-C linkages. They have useful mechanical properties and are widely used in industry. According to the current knowledge, these compounds are considered non-toxic since they are scarcely soluble in polar solvents. However, the question of whether they may accumulate in adipose tissue is still open.
2. The second compound class arises when vegetable oils are heated in the presence of oxygen. The evaluation of the formation mechanism is difficult because the majority of investigations have been performed using complex oil mixtures. It is not yet clear whether these are harmful compounds, but it is commonly accepted that simultaneously generated aldehydes (through scission at the double-bond position) are much more harmful [34]. This particularly applies if reaction products with amino groups (fried food) are considered.
3. The third class of FAHFAs is generated if a hydroxyl fatty acid reacts with a “normal” fatty acid. Their generation requires enzyme catalysis. The effects of FAHFAs as potential drugs are currently under intensive investigation. Several studies have provided convincing evidence that some FAHFAs possess antidiabetic and anti-inflammatory effects—but there may also be severe side effects. For instance, some selected FAHFAs induced hepatic steatosis and fibrosis in mice [64].
4. The last compound class has only been loosely investigated so far. These fatty acid oligomers are generated as the consequence of the oxidation/chlorination of unsaturated fatty acids such as oleic acid by HOCl. In contrast to all other compounds mentioned here, neither elevated temperatures nor enzymes nor any catalysts are necessary in vitro to generate oligomers from fatty acid chlorohydrins. This makes them interesting for many reasons. These compounds may be useful to monitor the in vivo generation of HOCl because they undergo a slower metabolic turnover in comparison to the native lipids [91].

Although the physiological effects of common “FAHFA” are obvious, the knowledge of the effects of oligomeric chlorohydrins is still limited because they are presumably generated under inflammatory conditions. Thus, further investigations to clarify these aspects are required, and we hope that this paper will foster the research interest.

## Data Availability

Not applicable.
